# Supplementary material for: Transcriptomic and Metabolomic Analyses of the Response of Resistant Peanut Seeds to Aspergillus flavus Infection
Source: Toxins (Basel). 2023 Jun 26;15(7):414. doi: 10.3390/toxins15070414 (PMC10467056; doi:10.3390/toxins15070414)
Supplement: Supplementary file 1 [file toxins-15-00414-s001.zip › Figure S1.pdf]

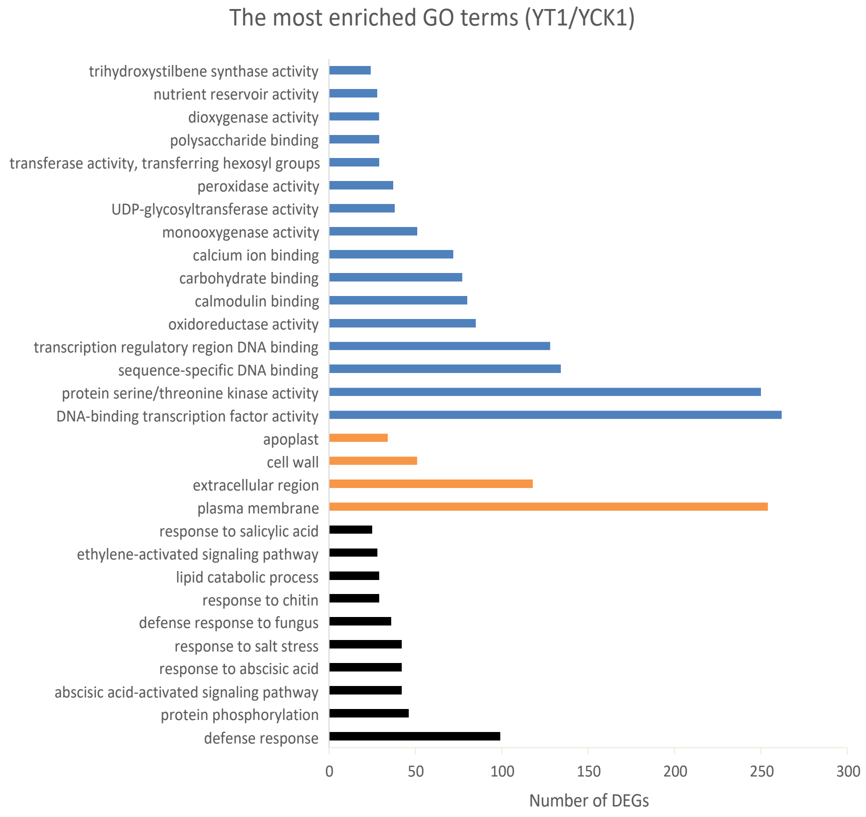

The most enriched GO terms (YT1/YCK1)
trihydroxystilbene synthase activity
nutrient reservoir activity
dioxygenase activity
polysaccharide binding
transferase activity, transferring hexosyl groups
peroxidase activity
UDP-glycosyltransferase activity
monooxygenase activity
calcium ion binding
carbohydrate binding
calmodulin binding
oxidoreductase activity
transcription regulatory region DNA binding
sequence-specific DNA binding
protein serine/threonine kinase activity
DNA-binding transcription factor activity
apoplast
cell wall
extracellular region
plasma membrane
response to salicylic acid
ethylene-activated signaling pathway
lipid catabolic process
response to chitin
defense response to fungus
response to salt stress
response to abscisic acid
abscisic acid-activated signaling pathway
protein phosphorylation
defense response
0
50
100
150
200
250
300
Number of DEGs

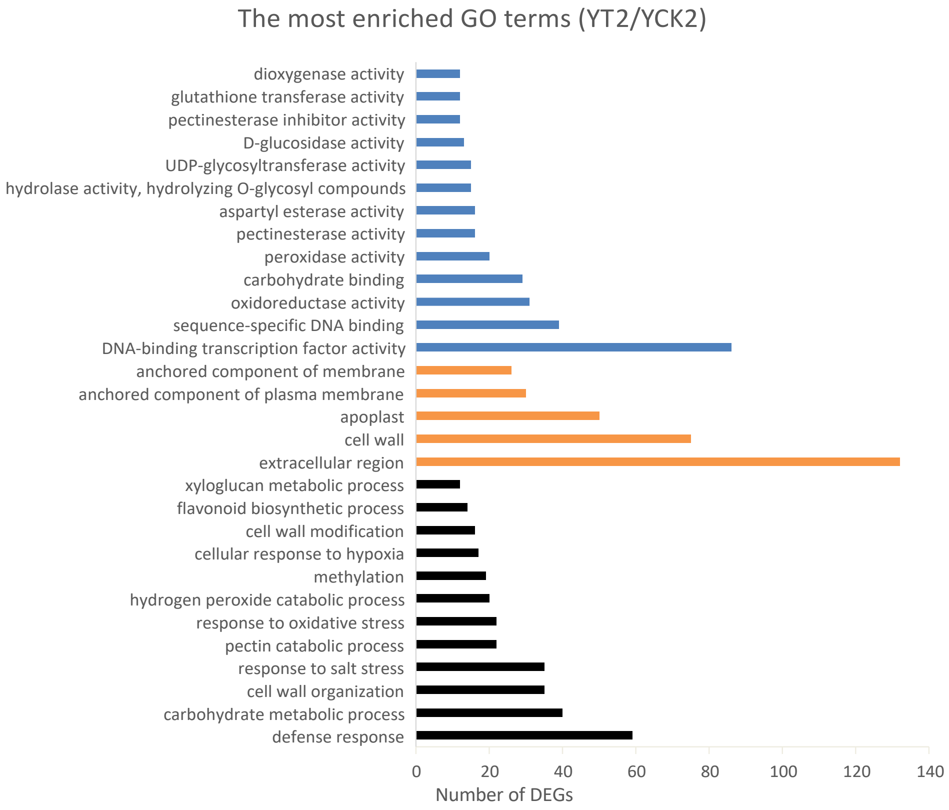

The most enriched GO terms (YT2/YCK2)
dioxygenase activity
glutathione transferase activity
pectinesterase inhibitor activity
D-glucosidase activity
UDP-glycosyltransferase activity
hydrolase activity, hydrolyzing O-glycosyl compounds
aspartyl esterase activity
pectinesterase activity
peroxidase activity
carbohydrate binding
oxidoreductase activity
sequence-specific DNA binding
DNA-binding transcription factor activity
anchored component of membrane
anchored component of plasma membrane
apoplast
cell wall
extracellular region
xyloglucan metabolic process
flavonoid biosynthetic process
cell wall modification
cellular response to hypoxia
methylation
hydrogen peroxide catabolic process
response to oxidative stress
pectin catabolic process
response to salt stress
cell wall organization
carbohydrate metabolic process
defense response
0
20
40
60
80
100
120
140
Number of DEGs

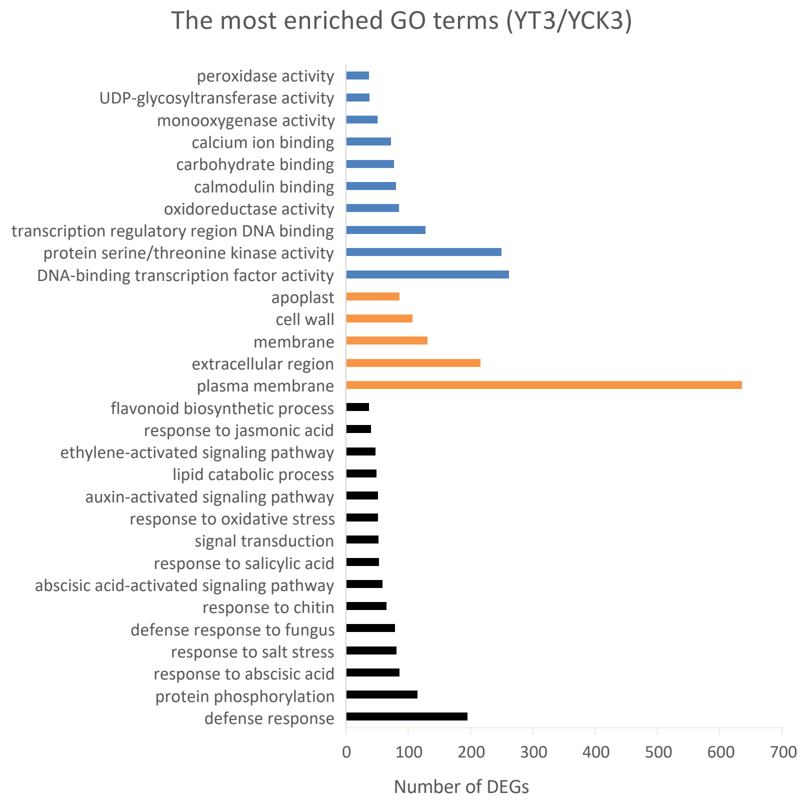
The most enriched GO terms (YT3/YCK3)
peroxidase activity
UDP-glycosyltransferase activity
monooxygenase activity
calcium ion binding
carbohydrate binding
calmodulin binding
oxidoreductase activity
transcription regulatory region DNA binding
protein serine/threonine kinase activity
DNA-binding transcription factor activity
apoplast
cell wall
membrane
extracellular region
plasma membrane
flavonoid biosynthetic process
response to jasmonic acid
ethylene-activated signaling pathway
lipid catabolic process
auxin-activated signaling pathway
response to oxidative stress
signal transduction
response to salicylic acid
abscisic acid-activated signaling pathway
response to chitin
defense response to fungus
response to salt stress
response to abscisic acid
protein phosphorylation
defense response
0 100 200 300 400 500 600 700
Number of DEGs

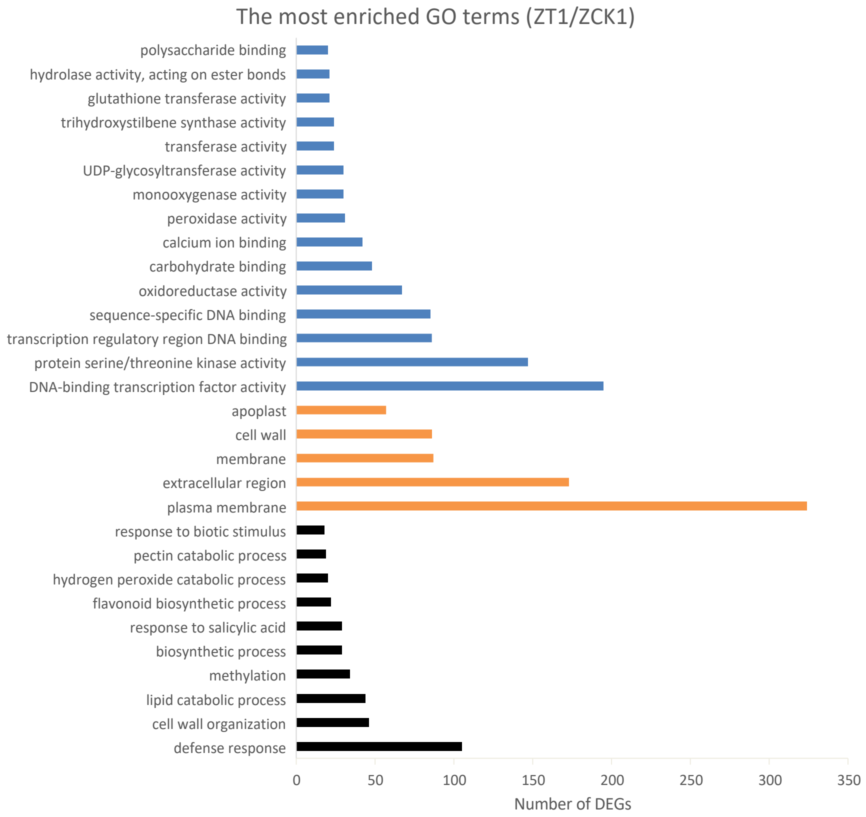
The most enriched GO terms (ZT1/ZCK1)
polysaccharide binding
hydrolase activity, acting on ester bonds
glutathione transferase activity
trihydroxystilbene synthase activity
transferase activity
UDP-glycosyltransferase activity
monooxygenase activity
peroxidase activity
calcium ion binding
carbohydrate binding
oxidoreductase activity
sequence-specific DNA binding
transcription regulatory region DNA binding
protein serine/threonine kinase activity
DNA-binding transcription factor activity
apoplast
cell wall
membrane
extracellular region
plasma membrane
response to biotic stimulus
pectin catabolic process
hydrogen peroxide catabolic process
flavonoid biosynthetic process
response to salicylic acid
biosynthetic process
methylation
lipid catabolic process
cell wall organization
defense response
0 50 100 150 200 250 300 350
Number of DEGs

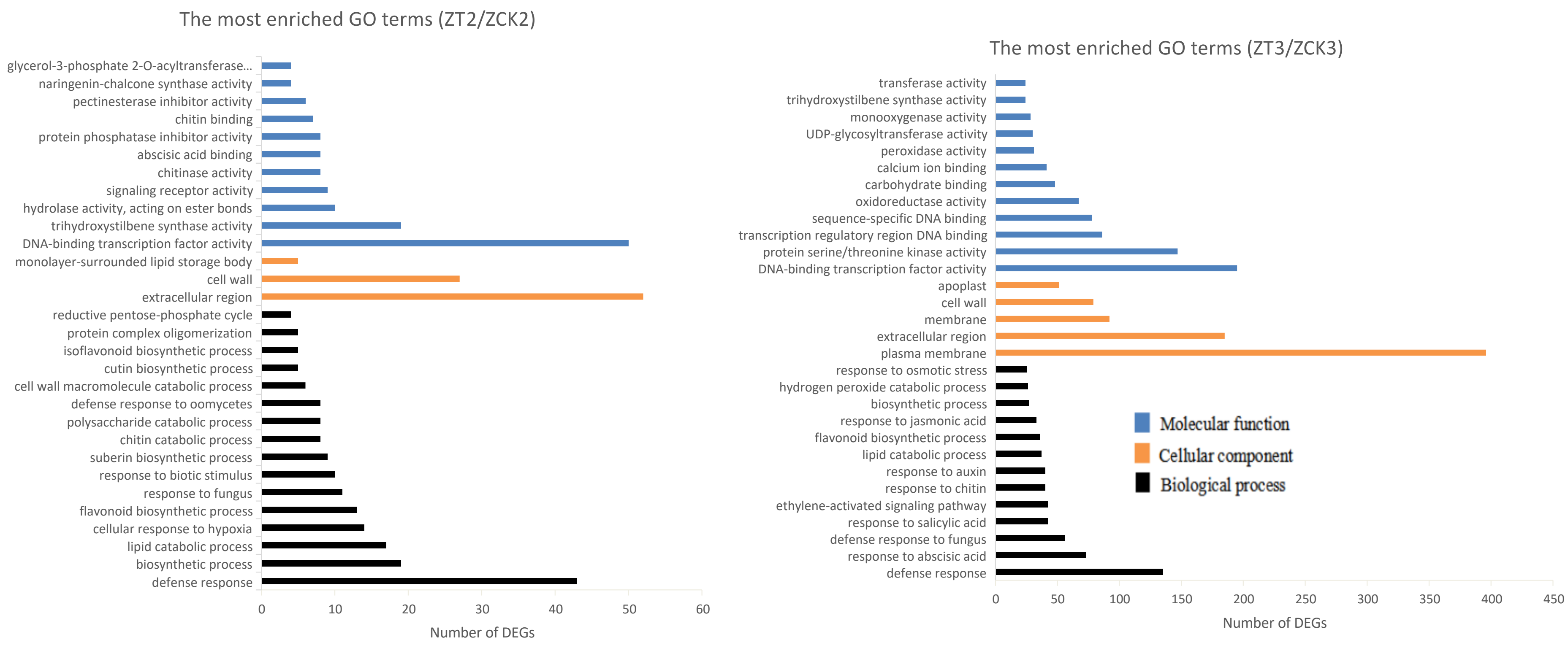

Figure S1: Top 30 GO term enriched functional categories Note: horizontal is number of DEGs， ordinate is GO term． Different colors are used to distinguish Biological Process(BP), Cellular Component(CC) and Molecular Function(MF)．
