# Supplementary material for: Transcriptomic and Metabolomic Analyses of the Response of Resistant Peanut Seeds to Aspergillus flavus Infection
Source: Toxins (Basel). 2023 Jun 26;15(7):414. doi: 10.3390/toxins15070414 (PMC10467056; doi:10.3390/toxins15070414)
Supplement: Supplementary file 1 [file toxins-15-00414-s001.zip › Figure S2.pdf]

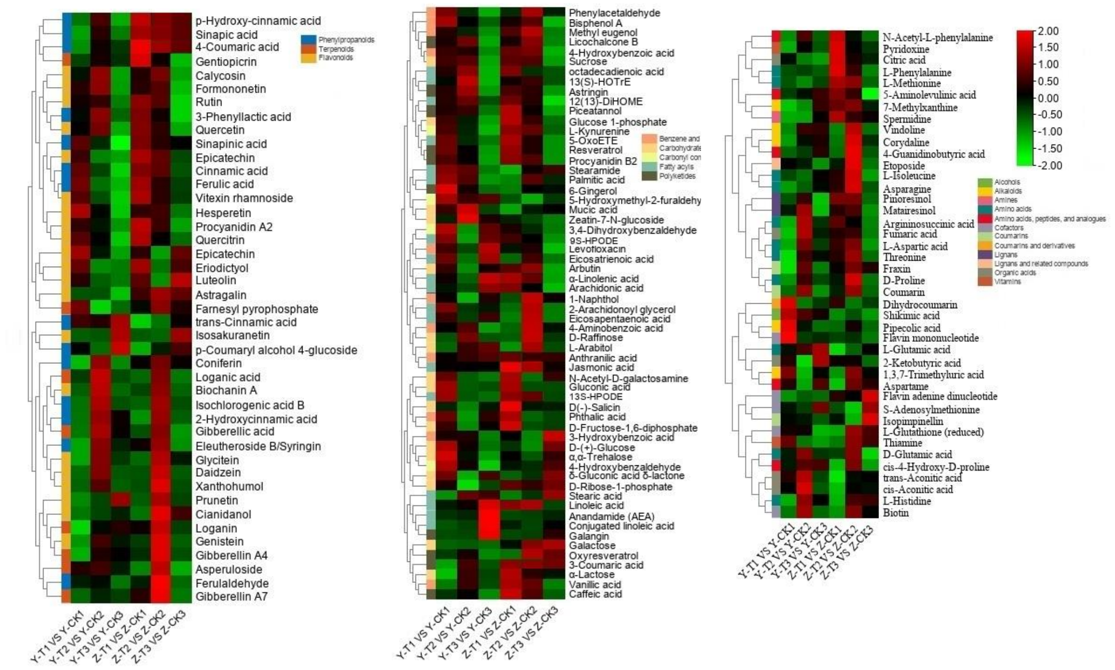

A B C

Figure S2: Heatmaps of differential metabolites: (A) Expression profiles of differential metabolic pathway of flavonoids、phenylpropanoids、Terpenoids.(B) Expression profiles of differential metabolic pathway of Benzene and derivatives、Carbohydrates、Carbonyl compounds、Fatty acyls、Polyketides. (C) Expression profiles of differential metabolic pathway of other differential metabolites.
