# Supplementary material for: Transcriptomic and Metabolomic Analyses of the Response of Resistant Peanut Seeds to Aspergillus flavus Infection
Source: Toxins (Basel). 2023 Jun 26;15(7):414. doi: 10.3390/toxins15070414 (PMC10467056; doi:10.3390/toxins15070414)
Supplement: Supplementary file 1 [file toxins-15-00414-s001.zip › Table S1.pdf]

Table S1: Primers used for analysis of gene expression by Real Time qRT-PCR

Primers	Sequences
13S-LOX(LOC107476778)-f	TCGTTGGTCGGAGAATAG
13S-LOX(LOC107476778)-r	GCGACGGAACCAGTATCA
4-coumarate--CoA ligase 2-like (LOC1074774 20) - f	AATGAGAAGGCGACAGCA
4-coumarate--CoA ligase 2-like (LOC1074774 20)-r	TCTTGAATGGAAGGGTGG
cellulose synthase(LOC107461063)-f	AACCACATTGCCAGAAGC
cellulose synthase(LOC107461063)-r	TTTGAAGTCCTGGGCTGA
chalcone synthase(LOC107483336)-f	ATGGTGGTTGTGGAGGTT
chalcone synthase(LOC107483336)-r	GAGTTTGGTGAGTCGGTA
chalcone--flavonone isomerase 1A (LOC107467625)-f	TTATGGCGACGCAGAAGC
chalcone--flavonone isomerase 1A (LOC107467625)-r	TGGCGGTAGAAGACGGAGG
chitinase(LOC107461673)-f	CGCAGTTTCTTCGCTCAC
chitinase(LOC107461673)-r	GCAAAGGTATCAGGGTCT
ethylene-responsive transcription factor ERF098(LOC107493082)-f	TCCGAATGTTATCTATCTG
ethylene-responsive transcription factor ERF098(LOC107493082)-r	AATCCCTCTGTAGTGTCTC
pathogenesis-related protein 2 (LOC107463224)-f	CGGAACCATCAAGAAACT
pathogenesis-related protein 2 (LOC107463224)-r	GAGCCACTCCTCCAACAA
Pectinesterase (LOC107475712)-f	GCGTTCTGGATCTGACTT
Pectinesterase (LOC107475712)-r	GTTTGGAGGGTTTCTTGC
peroxidase A2 (LOC107483153)-f	TAGCAGCAGTGGAAACCC
peroxidase A2 (LOC107483153)-r	TAGTCCCTGACCCACCAT
phenylalanine ammonia-lyase class 3 (LOC107496075)-f	ATCTCCCACCTACTCGTT
phenylalanine ammonia-lyase class 3 (LOC107496075)-r	CAGCCATCTTCCTTCCTA
stilbene synthase 3(LOC107482389)-f	TCGTACTCTTAGGGCTCG
stilbene synthase 3(LOC107482389)-r	ACCACGGAAAGTGATTGA
