# Supplementary material for: Transcriptomic and Metabolomic Analyses of the Response of Resistant Peanut Seeds to Aspergillus flavus Infection
Source: Toxins (Basel). 2023 Jun 26;15(7):414. doi: 10.3390/toxins15070414 (PMC10467056; doi:10.3390/toxins15070414)
Supplement: Supplementary file 1 [file toxins-15-00414-s001.zip › table S2.pdf]

Table S2: The quality assessment table of sequencing data and list of mapping results

sample name	total_raw _reads	total_clean_ reads	total_clean _bases	clean_read_ Q20	clean_read_Q 30	clean_reads_ ratio	total_mapping _gene_ratio	uniquely_mapping_ gene_ratio
Y_CK1	47.91	44.50	6.68	96.45	91.34	92.91	76.25	74.33
Y_CK2	47.91	44.53	6.68	96.43	91.30	92.97	74.14	72.02
Y_CK3	48.50	44.69	6.70	96.49	91.43	92.17	73.46	71.35
Y_T1	47.91	44.93	6.74	96.45	91.32	93.79	74.97	73.06
Y_T2	48.50	44.45	6.67	96.39	91.17	91.73	75.56	73.46
Y_T3	47.91	44.70	6.71	96.41	91.23	93.30	69.57	67.26
Z_CK1	47.33	44.32	6.65	96.49	91.38	93.64	76.21	74.24
Z_CK2	46.78	44.37	6.65	96.47	91.30	94.86	74.22	72.72
Z_CK3	47.91	44.62	6.70	96.34	91.06	93.14	74.29	72.31
Z_T1	47.33	44.41	6.66	96.90	92.17	93.83	72.46	70.24
Z_T2	47.33	44.43	6.66	96.94	92.24	93.87	74.56	71.53
Z_T3	47.33	44.20	6.63	96.90	92.13	93.40	73.50	
